# Comment on Early versus delayed mobilization for in-hospital mortality and health-related quality of life among critically ill patients: a systematic review and meta-analysis (Okada et al., *Journal of Intensive Care* 2019)

**DOI:** 10.1186/s40560-020-0436-7

**Published:** 2020-03-12

**Authors:** K. Friedrich Kuhn, Stefan J. Schaller

**Affiliations:** Charité – Universitätsmedizin Berlin, corporate member of Freie Universität Berlin, Humboldt-Universität zu Berlin, and Berlin Institute of Health, Department of Anesthesiology and Surgical Intensive Care, Berlin, Germany

**Keywords:** ICU, Critical illness, Early mobilization

## Abstract

Critical comment on the review by Okada et al. on the effect of early versus delayed mobilization because of their definition of early mobilization as mobilization within a week of ICU admission in contrast to current evidence.

## Comment

In their systematic review and meta-analysis, Okada et al. investigate the impact of early versus delayed mobilization for in-hospital mortality and health-related quality of life among critically ill patients, including 11 studies in their meta-analysis [1]. They compared randomized controlled trials (RCTs) starting mobilization within 1 week of ICU admission to those initiating mobilization later than 1 week.

Aware that there is no uniform definition of “early mobilization” in the ICU yet, to use 1 week as cut-off point seems unreasonable for various reasons. So far, only studies starting early mobilization within 72 h have been able to improve patient outcomes, as summarized in published narrative reviews [2] with adoption in practice guidelines [3]. Schweickert et al. applied physical therapy and interruption of sedation within 72 h of ICU admission causing higher independent functionality at hospital discharge, shorter duration of delirium, and more ventilator-free days [4]. In another single-center RCT, the effect of standardized rehabilitation therapy in patients with acute respiratory failure leads to functional results at 6 months after hospital discharge [5]. [6]. And the just published study of an early mobility program started within 48 h confirmed improvement in function and increased functional independence [6]. In contrast, studies starting mobilization later had no beneficial effect [2].

Another current meta-analysis using different definitions was able to show an effect of early mobilization [7]. Finally, Ding et al. showed in their network meta-analysis that initiation of mobilization within 48–72 h in mechanical ventilation patients may be optimal to improve intensive care unit-acquired weakness [8].

In conclusion, as timing seems crucial for patient-centered outcomes, early mobilization should be consistently defined as mobilization within 72 h of ICU admission.

## Data Availability

Not applicable
